# Characterizing the metabolic phenotype of intestinal villus blunting in Zambian children with severe acute malnutrition and persistent diarrhea

**DOI:** 10.1371/journal.pone.0192092

**Published:** 2018-03-02

**Authors:** Marta Farràs, Kanta Chandwe, Jordi Mayneris-Perxachs, Beatrice Amadi, John Louis-Auguste, Ellen Besa, Kanekwa Zyambo, Richard Guerrant, Paul Kelly, Jonathan Richard Swann

**Affiliations:** 1 Computational and Systems Medicine Group, Division of Surgery and Cancer, Imperial College, London, United Kingdom; 2 CIBER de Fisiopatología de la Obesidad y la Nutrición (CIBEROBN), Barcelona, Spain; 3 Tropical Gastroenterology & Nutrition group, University of Zambia School of Medicine, Lusaka, Zambia; 4 Barts & The London School of Medicine, Queen Mary University of London, London, United Kingdom; 5 University of Virginia, Charlottesville, VA, United States of America; Johns Hopkins Bloomberg School of Public Health, UNITED STATES

## Abstract

**Background:**

Environmental enteric dysfunction (EED) is widespread throughout the tropics and in children is associated with stunting and other adverse health outcomes. One of the hallmarks of EED is villus damage. In children with severe acute malnutrition (SAM) the severity of enteropathy is greater and short term mortality is high, but the metabolic consequences of enteropathy are unknown. Here, we characterize the urinary metabolic alterations associated with villus health, classic enteropathy biomarkers and anthropometric measurements in severely malnourished children in Zambia.

**Methods/Principal findings:**

We analysed 20 hospitalised children with acute malnutrition aged 6 to 23 months in Zambia. Small intestinal biopsies were assessed histologically (n = 15), anthropometric and gut function measurements were collected and the metabolic phenotypes were characterized by ^1^H nuclear magnetic resonance (NMR) spectroscopy.

Endoscopy could not be performed on community controls children. Growth parameters were inversely correlated with enteropathy biomarkers (*p* = 0.011) and parameters of villus health were inversely correlated with translocation and permeability biomarkers (*p* = 0.000 and *p* = 0.015). Shorter villus height was associated with reduced abundance of metabolites related to gut microbial metabolism, energy and muscle metabolism (*p* = 0.034). Villus blunting was also related to increased sucrose excretion (*p* = 0.013).

**Conclusions/Significance:**

Intestinal villus blunting is associated with several metabolic perturbations in hospitalized children with severe undernutrition. Such alterations include altered muscle metabolism, reinforcing the link between EED and growth faltering, and a disruption in the biochemical exchange between the gut microbiota and host. These findings extend our understanding on the downstream consequences of villus blunting and provide novel non-invasive biomarkers of enteropathy dysfunction. The major limitations of this study are the lack of comparative control group and gut microbiota characterization.

## Introduction

Nutritional disorders are glaring examples of health inequalities between high- and low-income countries, and within low-income countries. Malnutrition underlies almost half of all child deaths globally and contributes enormously to the unacceptably high under-5 mortality rates in many countries [1]. Chronic undernutrition is usually manifested as stunting (poor linear growth), affects 30–40% of children in Zambia [2], and is associated with increased mortality [3], reduced neurodevelopmental potential and decreased long-term economic productivity [4]. Chronic undernutrition can also result in severe enteropathy (environmental enteric dysfunction), which includes alterations to intestinal cell morphology, gut barrier disruption, and nutrient malabsorption. Another consequence of enteropathy is increased susceptibility to pathogenic infections, and poor response to oral vaccines and nutritional interventions. Acute malnutrition is usually manifest as wasting (loss of tissue), and severe acute malnutrition (SAM) carries the highest mortality [5], particularly if associated with complications. HIV has changed the epidemiology, pathogenesis and clinical presentation of SAM, and children with both conditions have a particularly high mortality [6]. Over the last two decades, developments in the approach to treatment such as standardized management protocols and ready to use therapeutic food (RUTF) have improved the outcome of SAM [7,8]. However, children with clinical complications of SAM requiring hospital treatment [9] often fail to respond to treatment [10], and continue to experience high mortality of up to 35% [5,11]. In our experience it is a subgroup of children with SAM and persistent diarrhoea who pose the most difficult management challenges, although the vast majority of children with SAM have a degree of enteropathy [11–13]. Currently, there is no perfect non-invasive biomarker for the diagnosis of enteropathy and there is a pressing need to greater understand the biochemical perturbations associated with enteropathy associated with SAM and diarrhea so that novel therapies can be devised and tested.

Metabolic profiling is a systems biology approach that simultaneously measures a comprehensive range of metabolites within a biological sample to capture the metabolic phenotype of the biological system. We have recently demonstrated the utility of studying the urinary metabolic phenotypes of children and rodents to understand the biochemical impact of malnutrition and stunting in children and rodents [14,15]. This allows the biomolecular mechanisms of developmental shortfalls to be studied as well as the elucidation of biomarkers to identify susceptible individuals. Importantly, metabolic profiles are the product of genetic and environmental factors, providing information on the interactions between the genotype, the diet, lifestyle, and gut microbiota. We postulated that a metabolic phenotyping approach may provide molecular insights into EED, so we set out to determine if ^1^H nuclear magnetic resonance (NMR) spectroscopy-based metabolic profiling approach could describe any of several facets of the EED disorder and find new EED biomarkers correlated with gut function.

## Methods

### Study patients

We studied children with persistent diarrhoea (three or more loose stools per day for 14 days or more) and severe acute malnutrition (SAM) in the malnutrition ward of the University Teaching Hospital, Lusaka, Zambia, which has been the setting for several previous studies [11,16]. In total, 20 children with SAM in hospital were studied. They were consecutive admissions to the ward where the inclusion criteria were fulfilled. SAM was defined as WLZ < -3 or MUAC < 11.5 cm or bilateral oedema. These children were severely malnourished but with no pathogen detected on first line investigations (stool microscopy and culture). In total 67% of the children has Kwashiorkor. Their management closely followed WHO guidelines [9] with standardised treatment protocols and universal administration of antibiotics and oral rehydration therapy. All children in the SAM group were receiving F100 milk-based formula in the few days leading up to the day of collection of these samples.

### Ethics statement

Children were recruited once stabilised, after the initial treatment phase when mortality is highest, by approaching the mother and following discussion with the whole family. A parent or guardian provided written informed consent on behalf of child participants. Approval to study these children was obtained from the University of Zambia Biomedical Research Ethics Committee (number 006-01-13, dated 15^th^ April 2013).

### Anthropometry

Length and weight were measured using a length board (Seca) and mother-and-child weighing scales. Mid upper arm circumference (MUAC) was measured in accordance with WHO standard protocols.

### Blood biomarkers

Following a three hour fast, blood (taken with 4% tetracaine topical anaesthesia) and urine samples were collected. Blood samples were collected into EDTA, plain and lithium heparin tubes (Vacutainer, Becton Dickinson, Plymouth, UK) and one additional lithium heparin tube (TekLab) for trace element analysis. Blood samples were kept in the dark for 20 minutes then centrifuged at 537 *g* for 15 minutes; serum was then aliquoted into 1.5 mL tubes for storage at -80°C. Quantification of lipopolysaccharide (LPS) was by the Pyrochrome Limulus Amoebocyte Lysate (LAL) assay (Associates of Cape Cod, Liverpool, UK). Serum Insulin like growth factor (IGF) 1, IGF binding protein 3 (IGFBP-3), LPS binding protein (LBP), soluble cluster of differentiation 14 (sCD14), cluster of differentiation 163 (CD163), fatty acid binding protein (FABP), and C reactive protein (CRP) were measured by ELISA (R&D systems, Abingdon, UK). GLP2 was measured by ELISA (Millipore, Watford, UK).

### Endoscopy and small intestinal biopsy

Endoscopy was carried out in a subsample of the study (n = 15) under sedation by an anaesthetist, using a Pentax EG-2490i paediatric gastroscope. Gastric acid was aspirated by syringe and pH tested using indicator paper (Sigma, Poole, UK) to within 0.5 pH units. Three biopsies were collected from the second part of the duodenum, and placed into saline prior to orientation under a dissecting microscope and fixation on cellulose acetate paper in formalin-saline. Morphometric parameters included measures of villus and crypt remodeling as reported in several previous studies [17–21]. Villus blunting is associated with reduced villus height, increased villus width, reduced villus epithelial surface, and increased villus compartment volume. Morphometry was carried out on digitized images of well-orientated biopsies, and measures of villus height (VH), villus width (VW), villus perimeter (VP), crypt depth (CD), and surface area:volume ratio obtained as previously described [19, 20]. At least six villus crypt units were measured in each case.

### Absorption and permeability testing

A test solution containing 0.2 g rhamnose, 1 g lactulose per dose (50 mL) was instilled directly into the duodenum at the end of the endoscopy, as previously described [19]. The median time to urine collection was 123 minutes (IQR 75–221) with a range from 34 to 314 minutes.

### ^1^H NMR spectroscopic analysis

Urine samples were collected from children prior to endoscopy, and immediately decanted into a container with 1 mL 0.5% chlorhexidine. These samples were stored at -80°C until transportation to London on dry ice. Urine samples (400 μL) were combined with 200 μL of phosphate buffer (pH 7.4; 100% D_2_O) containing 1 mM of 3-trimethylsilyl-1-[2,2,3,3-^2^H_4_] propionate (TSP) as an external standard and 2 mM sodium azide as a bacteriocide. Samples were mixed by vortexing and centrifugated at 10000 *g* for 10 minutes. The supernatant (550 μL) was transferred to a 5 mm internal diameter NMR tube. The metabolic profiles of the urine samples were measured by ^1^H nuclear magnetic resonance (NMR) spectroscopy using a 700 MHz Bruker NMR spectrometer operating at 300 K. For each urine sample a standard noesy experiment was performed with water-suppression using 8 dummy scans followed by 128 scans collected into 64K data points. A mixing time of 10 ms was used with an acquisition time of 3.8 s and a recycle delay of 3.0 s.

^1^H NMR spectra were manually corrected for phase and baseline distortions. Chemical shifts in the spectra were referenced to the TSP peak at δ 0.0 ppm. Spectra were digitized using an in-house Matlab (version R2012a, The Mathworks, Inc.; Natwick, MA) script. Resonances derived from water were removed to minimize distortions to the spectral baseline. Spectra were normalized to the total area.

### Statistical analysis

**Fig 1:**
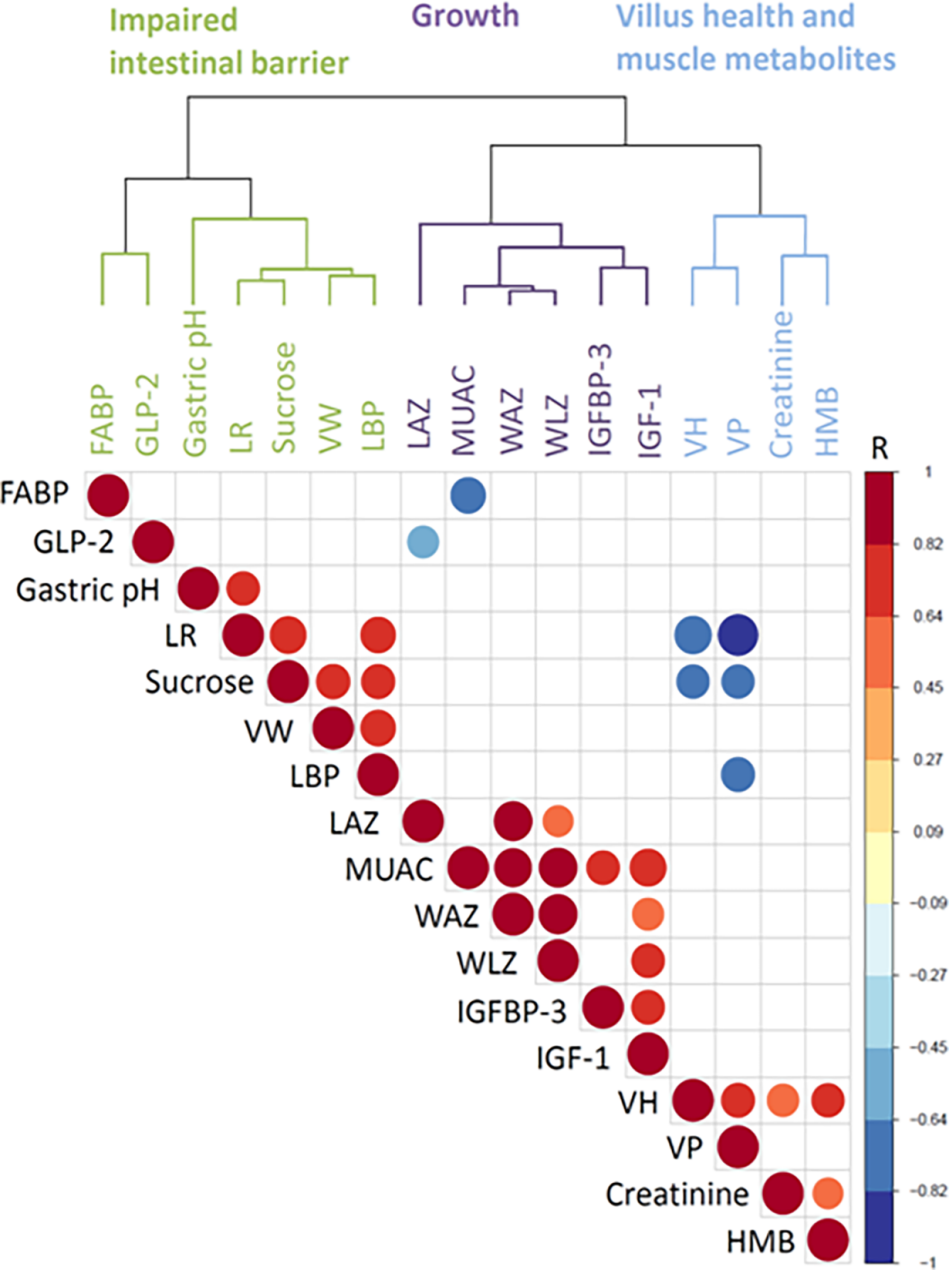
Correlodendogram with pearson correlations among bacterial translocation, intestinal permeability, villus structure, metabolites, and growth variables. Color intensity and size of the circle are proportional to the correlation coefficients. Significant correlations are shown following correction for multiple testing. FABP, fatty acid binding protein; LAZ, length-for-age Z score; IGF-1, insulin like growth factor 1; IGFBP-3, insulin like growth factor binding protein 3; LPS, lipopolysaccharide; LBP, lipopolysaccharide binding protein; MUAC, mid-upper arm circumference; VH, villus height; VPMM, villus perimeter or epithelial surface area; VW, villus weight; WAZ, weight-for-age Z score; WLZ, weight-for-length Z score.

Normality of continuous variables was assessed by normal probability plots. Non-normally distributed variables were log transformed if necessary. Pearson correlation analyses were used to evaluate relationships among variables. The significance level of the correlations was adjusted to p<0.017. This value of significance threshold (p = 0.017) (α) has been obtained by dividing the nominal significance threshold for a single test (c) by the number of effectively independent tests (n): α = c/n = 0.05/3 = 0.017. For the calculation of the n = 3 we used the hierarchical cluster analysis which was able to classify the 17 variables into three independent groups of biomarkers (gut function and inflammation, growth, and villus morphometry) (**Fig 1**). This method was adapted from Cheverud JM et al (2001) and is based on the variance and dissimilarity of the variables [22]. Furthermore, hierarchical clustering was used to order the variables and identify hidden structure within the data. Univariate statistical analyses were performed by SPSS 18.0 software (IBN Corp) and cluster/correlation analyses by R 3.2.1 (The R project).

Orthogonal projection to latent structures (OPLS) analysis with unit variance scaling was used to study the ^1^H NMR spectral profiles. Here, the metabolic profiles served as the descriptor (X) matrix and villus height (or classical measurements of gut health) was the response (Y) vector. The predictive ability of the model (Q^2^Y) was calculated using a seven-fold cross validation approach and its significance was determined by permutation testing (1000 permutations). Statistical analyses related to metabolomics were performed using an in-house Matlab (version R2012a, The Mathworks, Inc.; Natwick, MA) script.

## Results

### Anthropometry and growth factors biomarkers

Outliers were detected from the Box-plots in MUAC and FABP variables and eliminated from future analysis. These studied children were severely stunted (LAZ: mean = -3.06 ± 1.49), severely underweight (WAZ: mean = -3.49 ± 1.58), and moderately wasted (WLZ: mean = -2.68 ± 1.58) (**Table 1**). Correlation analysis found a direct association between IGF-1 and IGFBP-3 (r = 0.656; *p* = 0.011). IGF-1 was positively correlated with MUAC, and weight-for-height Z (WLZ) score (r = 0.789; *p* = 0.002; r = 0.654; *p* = 0.011, respectively) (**Fig 1**).

**Table 1:** Characteristics of the participants.

	Undernourished hospitalized children^a^ (n = 20)^b^
**GENERAL AND ANTHROPOMETRICS**	
**Sex: male**	11 (55%)
**Age** (months)	15.45 ± 5.42
**HIV: positive**	8 (40%)
**Mid-upper arm circumference (MUAC)** (cm)	11.14 ± 1.11
**Height for Age Z score (LAZ)**	-3.06 ± 1.49
**Weight for Age Z score (WAZ)**	-3.49 ± 1.58
**Weight for Height Z score (WLZ)**	-2.68 ± 1.58
**Insulin like growth factor 1 (IGF-1)** (ng/mL)	9.89 (9.25;14.28)
**Insulin like growth factor binding protein 3 (IGFBP-3)** (ng/mL)	736.74 ± 471.65
**ENTHEROPATIC AND PERMEABILITY BIOMARKERS**	
**Lactulose/Rhamnose ratio (L/R)**	0.38 ± 0.36
**Lipopolysaccharide (LPS)** (EU/mL)	522.72 ± 594.88
**LPS binding protein (LBP)** (ng/mL)	219.37 ± 107.71
**Glucagon-like peptide 2 (GLP-2)** (ng/mL)	2.75 ± 2.06
**Fatty acid binding protein (FABP)** (pg/mL)	2938.19 ± 1409.89
**CD163** (ng/mL)	1421.57 ± 843.26
**SCD14** (ng/mL)	2794.97 ± 1272.28
**C reactive protein (CRP)** (ng/mL)	3950.15 ± 5931.50
**GUT BIOPSY PARAMETERS**	
**Villus height (VH)** (μm)	197.94 ± 65.58
**Villus width (VW)** (μm)	148.01 (128.55;282.12)
**Crypt depth (CD)** (μm)	169.32 ± 31.91
**Villus perimeter (VP)** (μm)	451.65 ± 196.05

^a)^ Values expressed as mean ± S.D. or median (25th to 75th percentile).

^b)^ The sample size for gut biopsy parameters was 15.

### Measures of gut health

**Fig 2:**
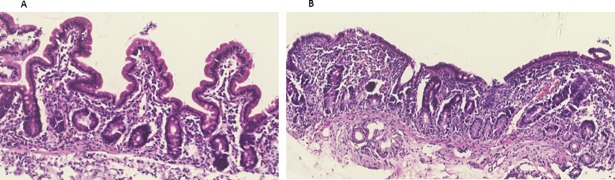
Mucosal abnormalities in biopsies from hospitalized children. The biopsies showed a range of abnormalities including (A) moderate villus blunting or (B) total villus atrophy.

Mucosal morphometry showed clear evidence of enteropathy with villus blunting and greatly increased inflammatory infiltrate (**Fig 2**) compared to literature controls (**S1 Table**). Measures of gut health also indicated villus blunting in the children studied (villus height (VH) mean = 197.94 ± 65.58 μm; villus perimeter (VP) mean = 451.65 ± 196.05 μm, **Table 1**) and villus inflammation (villus width (VW) median = 148.01 (128.55; 282.12) μm). Gut permeability was determined in the study children by measuring the lactulose:rhamnose ratio (LR; mean 0.38 ± 0.36) in urine and gut microbial translocation was evaluated by measuring serum LPS binding protein (LBP) (mean = 219.37 ± 107.71 μg/mL).

Correlation analysis revealed statistical associations between several villus morphometry parameters and measures of gut function (**Fig 1**). VH and VP were positively correlated (r = 0.67; *p* = 0.007) as were LR and LBP (r = 0.73; *p* = 0.016). An inverse relationship was seen between the LR and VP (r = -0.95; *p* = 0.000) and LR and VH (r = -0.81; *p* = 0.015). LBP was positively associated with VW (r = 0.73; *p* = 0.016). Furthermore, fatty acid binding protein (FABP) was negatively correlated with MUAC (r = -0.73; *p* = 0.011).

Non-significant correlations were observed among CD14, CD163, CRP, gastric pH, GLP2, and LPS variables neither with other parameters.

### Urine metabolic signatures associated with villus height

**Fig 3:**
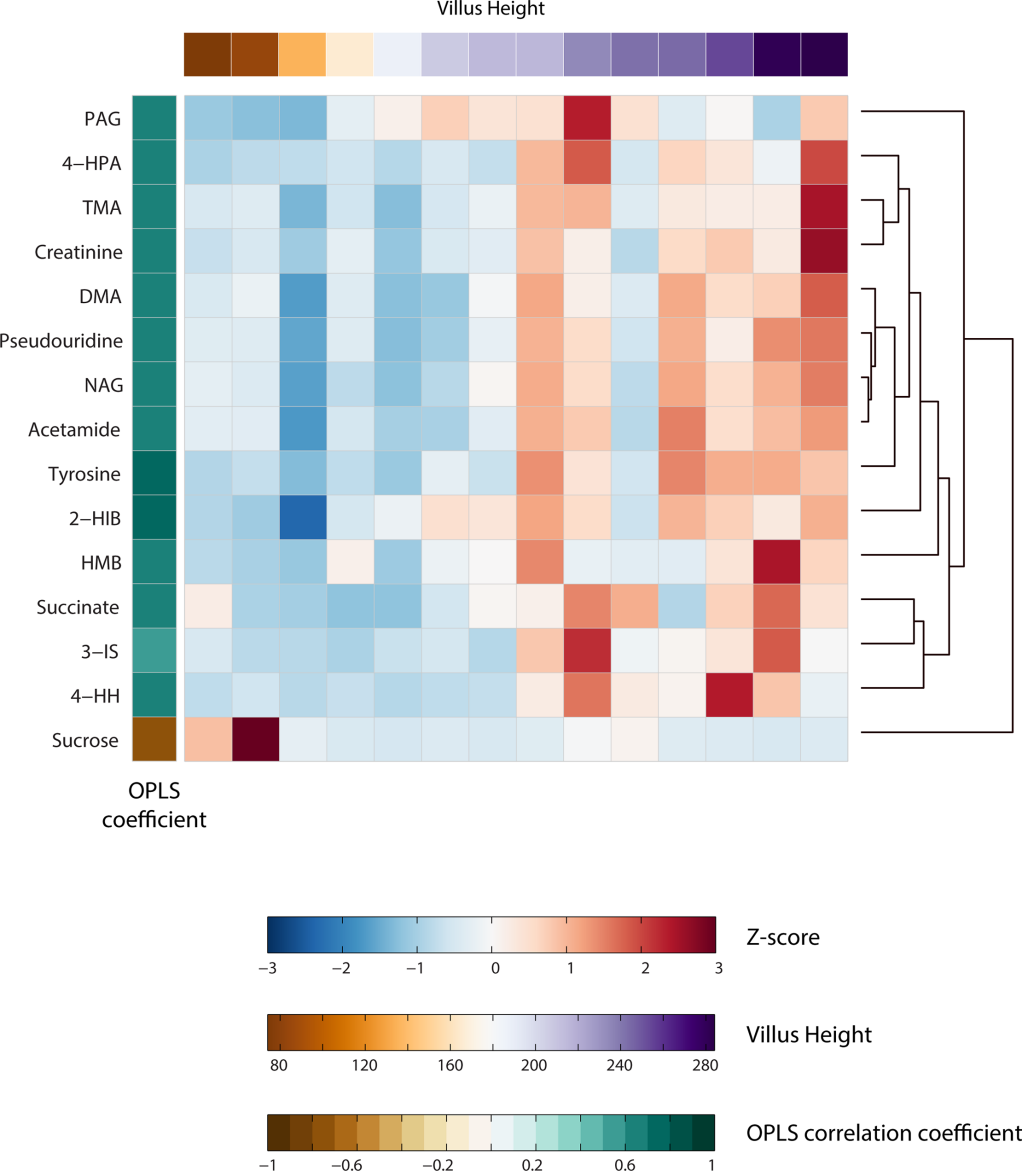
Dendogram and heatmap representation of unsupervised hierarchical clustering (HCA) of the metabonome for all children sorted according to the villus height (VH). Each column corresponds to a single children and each row corresponds to a specific metabolites. Only metabolites identified to correlate with VH through and OPLS model were used for metabolite clustering. Metabolite z-score transformation was performed on the levels of each metabolite across samples, with blue denotating a lower and red a higher level compared to the mean. Metabolites were clustered using correlation distance and average linkage. DMA, dimethylamine; 2-HIB, 2−hydroxyisobutyrate; 4-HPP, 4-hydroxypurate; 4-HPA, 4-hydroxyphenylacetate; 3-IS, 3−indoxyl sulfate; NAG, N-acetylglycoprotein; PAG, phenylacetylglutamine; TMA, trimethylamine.

An outlier was detected from the PCA analysis and eliminated from future analyses. A significant OPLS model was obtained identifying metabolic variation associated with VH (Q^2^Y = 0.303; *p* = 0.034). Metabolites found to be significantly correlated with VH in this model are visualized in a clustergram in **Fig 3**. Here, the *z*-scores of the peak integrals for the metabolites are plotted for each child along with the correlation coefficients extracted from the OPLS model. Metabolites related to gut microbial metabolism were excreted in lower amounts by individuals with shorter VH. This included 2−hydroxyisobutyrate (2-HIB), 4−hydroxyphenylacetate (4-HPA), phenylacetylglutamine (PAG), 3−indoxyl sulfate (3-IS), acetamide, 4−hydroxyhippurate (4-HH), and microbial metabolites of choline (dimethylamine (DMA), trimethylamine (TMA)). Metabolites related to energy and muscle metabolism (succinate, creatinine, β-hydroxy-β-methylbutyrate (HMB)) were also excreted in lower amounts by children with blunted villi (shorter VH) as were tyrosine, pseudouridine, and *N*-acetyl-glycoprotein (NAG). Increased excretion of sucrose was associated with villus blunting although this was driven by the large excretion of sucrose by two individuals with the shortest villi. Additional OPLS models were built using the other measures of gut health and classical measures of EED and growth as the response vector, however, no other significant OPLS models were obtained (**S2 Table**).

As markers of muscle mass, creatinine and HMB, were correlated with other measures of growth and gut health and function (**Fig 1**). Creatinine and HMB were positively correlated with each other (r = 0.55; *p* = 0.016). HMB was positively correlated with VH (r = 0.64; *p* = 0.013). Creatinine was also positively correlated to VH reaching a borderline significance (r = 0.61; *p* = 0.022). Interestingly, creatinine and HMB were found to cluster with villus health parameters (VH and VP). In addition, sucrose was found to cluster with measures of impaired intestinal barrier function. Furthermore, sucrose was positively correlated with LR, LBP, and VW, and negatively correlated with VH and VP (r > 0.67; p < 0.017) (r = -0.64; p <0.017).

## Discussion

The lack of significant progress in reducing mortality in the most severely ill children with SAM and intestinal disease over the last few decades requires fresh approaches. Here we report a study of the metabolome in 20 hospitalized children with SAM and diarrhoea, in our experience the group with the worst outcome, in order to identify urinary and plasma biomarkers of enteropathy. An integrated multi-tier approach was used combining morphological assessments of gut health using intestinal biopsy samples with anthropometric and biochemical measures of gut function, growth and metabolic status. We have identified signatures of disturbed metabolism of the gut microbiota, notably choline metabolism, and reduction of several specific host metabolic pathways including energy and muscle metabolism as well as tyrosine, pseudouridine, and *N*-acetyl glycoprotein. In contrast, sucrose excretion was increased with villus blunting.

Environmental enteric dysfunction arises from a vicious cycle of undernutrition and enteric infections. It is characterized by villus blunting, loss of barrier function (leaky gut), and malabsorption and has been implicated in growth stunting and cognitive impairments [23,24]. Intestinal biopsies collected in this study indicated that all children studied exhibited severe enteropathy with blunted villi (VH: 197.94 ± 65.58 μmm), severe enteric dysfunction (L/R ratio: 0.38 ± 0.36, LBP: 219.37 ± 107.71 μg/mL) and inflammation (CRP: 3950.15 ± 5931.50 ng/mL). Reductions in epithelial surface area, measured as VP, have been previously observed in Zambian adults with EED [20], and we found evidence that this could be an adaptation process to reduce microbial translocation.

Correlation analysis identified a statistical relationship between altered morphological structure and disrupted function with an inverse correlation between VH (positive correlation with villus blunting) and small intestinal permeability (LR ratio). VW, another parameter associated with villus blunting, showed a positive correlation with LBP linking villus atrophy with bacterial translocation. LBP, LR, and LPS in our studied children with SAM were higher than in community children who were not acutely malnourished [21]. Furthermore, in the studied children, LPS was two orders of magnitude higher than in Italian children with fatty liver disease [25] and higher than in adults with EED [20]. As well as functional consequences associated with morphological changes in the gut several markers of gut health/function were also associated with growth. FABP, a marker of enterocyte death and gut barrier disruption, was negatively correlated with MUAC, a key measure of growth. Similar observations have been reported in a cohort of community children with EED in Brazil [26]. As expected, MUAC was positively associated with other measures of growth (WAZ, WLZ) and promoters of growth (IGF-1).

Metabolic phenotyping was applied to characterize the urinary metabolic profiles of these children to better understand the biochemical mechanisms underlying growth shortfalls resulting from impaired gut health. Using a high-resolution untargeted ^1^H NMR spectroscopy based approach we found numerous urinary metabolites strongly correlated with villus height which is currently the gold standard for defining enteropathy. Villus blunting was associated with a lower excretion of creatinine and HMB, metabolites related to muscle metabolism and anabolism, indicating that muscle mass is reduced with villus blunting. Consistent with this observation, we have recently reported a reduction in creatinine excretion in undernourished Brazilian children with impaired growth [15]. HMB is a metabolite of leucine and reduced excretion of HMB with blunted villi may reflect impaired uptake of this branched chain amino acid from the diet. Muscle loss results from an imbalance between muscle protein synthesis and muscle protein degradation. HMB has been found to stimulate muscle protein synthesis through the mTOR-p70S6K1 pathway [27]. Moreover, the ubiquitin-proteasome proteolytic pathway is responsible for increased protein degradation in many disorders and can be induced by the pro-inflammatory cytokine tumor necrosis factor- α (TNF-α) following LPS exposure. Interestingly, HMB has been shown to attenuate the increase in protein degradation induced by TNF-α [28]. Furthermore, when given in combination with L-arginine and L-glutamine, HMB has been demonstrated to prevent lean tissue loss in patients with AIDS-associated wasting [29]. As such, further work is required to greater understand the multiple factors driving muscle loss with SAM and to evaluate the potential for HMB or leucine supplementation to ameliorate such effects.

In this study, urinary excretion of sucrose was associated with a shorter villus height. The two children with the most blunted villi drove these observations although comparisons with healthy children are not possible. In health, sucrose is derived from the diet and is hydrolyzed by sucrase in the brush border to glucose and fructose. In our unit, children with SAM are treated with standard nutritional rehabilitation which includes formula feeds containing sucrose. Increased sucrose excretion with blunted villi suggests that urinary sucrose is not sufficiently broken down when the mucosa is damaged and that paracellular absorption occurs following a loss of barrier function. This is supported by the positive correlation between sucrose excretion and LBP and LR. Sucrose excretion could be used as a diagnostic test to evaluate barrier function without the need for prior consumption of test sugars (*e*.*g*. lactulose, rhamnose, mannitol). However, further work is required to evaluate the mucosal damage threshold at which sucrose is absorbed. Previous studies by other groups discounted the sucrose permeation test as a test of small intestinal permeability [30–32], but sucrose excretion in urine may reflect the more extreme enteropathy in SAM.

Reductions in epithelial surface area seen in these children have been previously observed in Zambian adults with EED. This might represent an adaptive response to reduce microbial translocation [20]. Although the composition or abundance of the gut microbiota has not been measured in these children, our approach does enable to functional state of the microbiome to be characterized. Villus blunting was associated with a reduced excretion of gut bacterial-host co-metabolites. This included a range of metabolites derived from the bacterial breakdown of amino acids including tryptophan (3-IS, acetamide), phenylalanine (PAG, 4-hydroxyphenylacetate), as well as bacterial metabolism of polyphenols (4-hydroxyhippurate), choline (DMA, TMA), and carbohydrates (2-hydroxyisobutyrate). A reduction in epithelial surface area, or a change in the abundance or composition of mucins, would decrease availability of binding sites for community assembly. It is feasible that this may reduce or modify the microbial populations present in the gut and reduce the flow of microbial products to the host. In agreement with this, compositional and functional variation in the gut microbiota has been previously observed with undernutrition in rodent and human studies [14,33,34,35]. However, the reduced excretion of microbial-derived metabolites observed with villus blunting may also be driven by reduced uptake of microbial products due to malabsorption. As such, further studies are necessary to characterize the gut microbial changes associated specifically with villus blunting to clarify this observation.

No gold standard biomarker for EED currently exists and an invasive gut biopsy is required for a definitive diagnosis. In this study, we have combined intimate measures of gut health with information-rich metabolic phenotypes to illuminate novel urinary biomarkers indicative of specific gut damage. To this effect, several biomolecular features were discovered to be associated with villus height. This panel of non-invasive biomarkers included metabolites from endogenous and exogenous sources and warrant further exploration. A major limitation of this work is the lack of a comparative control group studying children from the same setting with a healthy gut. However, as an endoscopy could not be performed on community control children this was not possible. In addition, due to the intensive and time-consuming nature of the procedure used the sample size was relatively small. While a larger sample size may have yielded additional biomarkers of gut health, the detection of the current biomarkers in this smaller group indicates the significance of their relationship with villus height.

## Conclusions

To the best of our knowledge, this is the first time that morphological assessments of gut health have been correlated with non-invasive measures of growth, gut health and metabolic status. These findings imply that structural alterations induced by the chronic cycle of undernutrition and infection impact on the functioning of the gut with a downstream impact on protein synthesis, muscle development, and ultimately growth. Furthermore, modifications to the villus environment also modulate the important relationship between the intestinal microbiota and the host. These finding brings new knowledge to EED and may lead to non-invasive biomarkers of enteropathy.

## Supporting information

S1 Table Comparison of morphometric measurements with historical data. VH, villus height; CD, crypt depth; VH/CD, villus height/crypt depth.
(DOCX)

S2 Table Summary of the orthogonal projection to latent structures (OPLS) models returned for the various measures. Q_2_Y, the goodness of prediction.
(DOCX)

## Abbreviations

CD: crypt depth
CD163: cluster of differentiation 163
DMA: dimethylamine
EED: environmental enteric dysfunction
FABP: fatty acid binding protein
LAZ: length-for-age Z score
^1^H NMR: proton nuclear magnetic resonance
IGF-1: insulin like growth factor 1
IGFBP-3: insulin like growth factor binding protein 3
LPS: lipopolysaccharide
LBP: lipopolysaccharide binding protein
MUAC: mid-upper arm circumference
NAG: N-acetylglycoprotein
NMNA: N-methylnicotinamide
OPLS: orthogonal projection to latent structures
PAG: phenylacetylglutamine
SAVR: villus surface area:volumen ratio
sCD14: soluble cluster of differentiation 14
TMA: trimethylamine
VH: villus height
VP: villus perimeter or epithelial surface area
VW: villus weight
WAZ: weight-for-age Z score
WLZ: weight-for-length Z score
