# Correction: Characterizing the metabolic phenotype of intestinal villus blunting in Zambian children with severe acute malnutrition and persistent diarrhea

**DOI:** 10.1371/journal.pone.0196934

**Published:** 2018-05-01

**Authors:** The PLOS ONE Staff

The following grant is missing from the Funding section: Gates Foundation (OPP1066118).

The publisher apologizes for the error.
