# Supplementary material for: Characterizing the metabolic phenotype of intestinal villus blunting in Zambian children with severe acute malnutrition and persistent diarrhea
Source: PLoS One. 2018 Mar 2;13(3):e0192092. doi: 10.1371/journal.pone.0192092 (PMC5834158; doi:10.1371/journal.pone.0192092)
Supplement: S1 Table — VH, villus height; CD, crypt depth; VH/CD, villus height/crypt depth. (DOCX) [file pone.0192092.s001.docx]

**S1 Table. Comparison of morphometric measurements with historical data.**

| Reference | Sample description | VH ^a^ *(µm)* | CD ^a^ *(µm)* | VH/CD ^a^ |
| --- | --- | --- | --- | --- |
| Penna et al^1^ | British children *n=24* | 332 (45) | 169 (28) | 2.00 (0.35) |
| Campbell et al^2^ | British children *n=19* | 355 (35) | 170 (20) | 2.1 (0.3) |
| Cook et al^3^ | Ugandan children  (Kwashiorkor 4 years previously) | 321 [271-359] | - | - |
| Gendrel D^4^ | Gambian children with SAM *n=13* | 218 (43) | 154 (17) | - |
| Gendrel D^4^ | Gambian children with malnourished *n=38* | 243 (68) | 278 (69) | - |

^a)^ Values expressed as mean ± S.D. or median [range].

[1] Penna FJ, Hill ID, Kingston D, Robertson K, Slavin G, Shiner M. Jejunal mucosal morphometry in children with and without gut symptoms and in normal adults. *J Clin Path* 1981; 34: 386-392.

[2] Campbell DI, Murch SH, Elia M, et al. Chronic T cell-mediated enteropathy in rural west African children: relationship with nutritional status and small bowel function. *Pediatr Res* 2003; 54: 306-11.

[3] Cook GC, Lee FD. The jejunum after kwashiorkor. Lancet 1966; ii: 1263-1267.

[4] Gendrel D, Gahouma D, Ngou-Milama E, Nardou M, Chamlian A, Philippe E. Anomalies de la muqueuse jejunale et malnutrition protein-calorique chez le nourrisson en Afrique equatorial. *Ann Pediatr* 1984; 31: 871-876.
