# Supplementary material for: Characterizing the metabolic phenotype of intestinal villus blunting in Zambian children with severe acute malnutrition and persistent diarrhea
Source: PLoS One. 2018 Mar 2;13(3):e0192092. doi: 10.1371/journal.pone.0192092 (PMC5834158; doi:10.1371/journal.pone.0192092)
Supplement: S2 Table — Q2Y, the goodness of prediction. (DOCX) [file pone.0192092.s002.docx]

**S2 Table. Summary of the orthogonal projection to latent structures (OPLS) models returned for the various measures.**

| Variable | N | Q_2_Y | p-value |
| --- | --- | --- | --- |
| Age | 19 | -2.539 | - |
| Sex | 19 | 0.144 | 0.083 |
| HIV | 19 | -1.492 | - |
| MUAC | 1~~8~~5 | ~~-0.209~~ 0.003 | 0.314 |
| LAZ | 19 | -0.187 | - |
| WAZ | 19 | -0.192 | - |
| WLZ | 19 | -0.253 | - |
| IGF-1 | 13 | -0.752 | - |
| IGFBP-3 | 13 | -0.578 | - |
| LR | 10 | -0.604 | - |
| LPS | 13 | -0.163 | - |
| LBP | 13 | -0.431 | - |
| GLP-2 | 12 | -0.211 | - |
| FABP | 1~~3~~2 | ~~-0.568~~ -0.370 | - |
| CD163 | 13 | -1.057 | - |
| sCD14 | 13 | -0.885 | - |
| CRP | 13 | -0.554 | - |
| VH | 14 | 0.303 | 0.034 * |
| VW | 14 | -3.624 | - |
| CD | 14 | -1.135 | - |
| VP | 14 | -4.130 | - |

- p < 0.05 was considered significant.
